# The reliability of toe systolic pressure and the toe brachial index in patients with diabetes

**DOI:** 10.1186/1757-1146-3-31

**Published:** 2010-12-22

**Authors:** Mary T Romanos, Anita Raspovic, Byron M Perrin

**Affiliations:** 1 Department of Podiatry and Musculoskeletal Research Centre, Faculty of Health Sciences, La Trobe University, Bundoora, Victoria, 3086, Australia; 2 La Trobe Rural Health School and Musculoskeletal Research Centre, Faculty of Health Sciences, La Trobe University, PO Box 199, Victoria, 3552, Australia

## Abstract

**Background:**

The Ankle Brachial Index is a useful clinical test for establishing blood supply to the foot. However, there are limitations to this method when conducted on people with diabetes. As an alternative to the Ankle Brachial Index, measuring Toe Systolic Pressures and the Toe Brachial Index have been recommended to assess the arterial blood supply to the foot. This study aimed to determine the intra and inter-rater reliability of the measurement of Toe Systolic Pressure and the Toe Brachial Index in patients with diabetes using a manual measurement system.

**Methods:**

This was a repeated measures, reliability study. Three raters measured Toe Systolic Pressure and the Toe Brachial Index in thirty participants with diabetes. Measurement sessions occurred on two occasions, one week apart, using a manual photoplethysmography unit (Hadeco Smartdop 45) and a standardised measurement protocol.

**Results:**

The mean intra-class correlation for intra-rater reliability for toe systolic pressures was 0.87 (95% LOA: -25.97 to 26.06 mmHg) and the mean intra-class correlation for Toe Brachial Indices was 0.75 (95% LOA: -0.22 to 0.28). The intra-class correlation for inter-rater reliability was 0.88 for toe systolic pressures (95% LOA: -22.91 to 29.17.mmHg) and 0.77 for Toe Brachial Indices (95% LOA: -0.21 to 0.22).

**Conclusion:**

Despite the reasonable intra-class correlation results, the range of error (95% LOA) was broad. This raises questions regarding the reliability of using a manual sphygmomanometer and PPG for the Toe Systolic Pressure and Toe Brachial Indice.

## Background

The prevalence of diabetes is increasing with peripheral arterial collusive disease (PAOD) being a common condition in this population [1-4]. PAOD is a progressive disorder that affects approximately twenty five per cent of adults in Australia who are over 55 years of age or have diabetes [5]. The risk of PAOD is increased, it occurs earlier and is often more aggresive and diffuse in patients with diabetes, particularly targeting the distal popilteal and trifurcation vessels [6-10]. Despite the established relationship between PAOD and diabetes, PAOD is still largely under-diagnosed and undermanaged in this population [11]. This may be due to the reduced diagnostic utility of traditional assessments in diabetes. Mönckeberg's sclerosis causes incompressibility of arteries in this population, which may affect the accuracy of Ankle Brachial Indices (ABI) by falsely elevating the measurement. There is a need for reliable and valid non-invasive assessment tools to enhance the clinical assessment for PAOD in people with diabetes.

The Australian Diabetes Society recommends that vascular screening in people with diabetes be performed annually for early diagnosis of PAOD to enable risk reduction strategies to be implemented [3]. There is debate regarding which assessment method is most effective for diagnosis [12-14]. The assessment of peripheral vascular status in a clinical setting includes questioning and clinical examination, combined with a variety of tests such as the Ankle Brachial Index (ABI) and Toe Brachial Index (TBI). The ABI is a very useful clinical test to assess the arterial blood supply to the foot, but there are limitations to this method when conducted on people with diabetes [8,15,16]. Medial calcification in diabetes, known as Mönckeberg's sclerosis, causes the hardening and incompressibility of arteries which can affect the accuracy of ABIs [17,18]. The hardening of the artery is due to to the stiffening of the elastic layer of the arterial wall, but in contrast to intimal artery calcification, it does not obstruct the arterial lumen [19]. In addition Mönckeberg's sclerosis is highly prevalent in autonotmic neuropathy and chronic renal insufficiency [19]

As an alternative, toe systolic pressures and/or TBIs have been recommended as they are reported to be less affected by medial calcification [8,20-24] and false positive results are reported to be rare [8,20,25]. The Second European Consensus Document and the Trans-Atlantic Inter-Society Consensus recommends an absolute toe pressure of <30 mmHg when defining critical ischemia [26,27]. These recommendations indicate the toe systolic pressure and the TBI to be useful as they can predict outcomes and are less affected by the presence of medial calcification. In recent years, there has been an increase in opportunity to measure toe systolic pressures and TBIs in general practice, with the equipment used to take such measures becoming more affordable.

There is limited research exploring the reliability of toe systolic pressures and TBIs in patients with diabetes, particularly with respect to the newer, more affordable devices. Some research has explored reliability of the measurement of Toe Systolic Pressures in patients with diabetes and varying stages of PAOD with intra-class correlations (ICCs) ranging from 0.77 to 0.99 in intra-rater reliability [28-31] and 0.85 to 0.93 in inter-rater reliability [29,31] (Table 1). De Graaff and colleagues assessed the reliability of toe systolic pressures in 60 patients with 36% with diabetes [21]. They reported the reliability of toe systolic pressures across 2 test sessions to be substantial; however the absolute variation was larger than predicted (15%) [22]. Cloete et al. investigated the intra-rater reliability of the toe systolic pressure in patients with known PAOD, carotid artery disease but not history of PAOD and control patients [23]. All measurements were made by a single vascular technologist.

**Table 1:** Comparison of results gained from previous studies measuring toe systolic pressures

Study	Sample size	Results
de Graaff, et al. (2000) [28]	n = 60	ICC = 0.92 for intra-rater

de Graaff, et al. (2001) [29]	n = 54	ICC = 0.92 for intra-rater

Cloete, et al (2009) [30]	n = 50	ICC = 0.85 for inter-rater ICC = 0.77-0.99 for intra-rater

Scanlon, et al (2009) [31]	n = 60	ICC = 0.78-0.79 for intra-rater ICC = 0.93 for inter-rater

Note: ICC = Intra-class coefficient.

One study has investigated the reliability of the measurement of TBIs [31]. The results showed intra and inter-rater reliability ICCs of 0.51 to 0.72 and 0.85 respectively although the study is yet to be published. In this particular study, an automatic photoplethsmography (PPG) system was used to obtain the systolic pressures; the reliability was not investigated using a manual PPG unit. Additional points with the past research include difficulty in interpreting the results as the error range in the units of measurement were not reported in most of the studies [28-30] The methodology and protocol were briefly explained and only intra-rater reliability was investigated in two of the studies [28,30].

Toe systolic pressures has been available since the early 1930s and recommended in patients with PAOD and a fasely elevated ABI [8]. Although, the toe systolic pressure can be measured in the clinical setting using a PPG, it has not been widely available or routinely performed in general clinical practice as it can be expensive and there is limited research investigating the reliability and validity of this measurement [11]. In recent years, portable continuous wave Doppler units have been used to measure toe systolic pressures when the ABI is elevated. However when the toes are cold, Doppler-derived toe systolic pressure are unreliable due to vasoconstriction of digital arteries. This effect persists even when attempts are made to control the temperature of the testing environment [15]. Therefore a low toe systolic pressure may be associated with PAOD or vasoconstriction of the arteries [15]. Toe systolic pressures obtained via PPG are yet to be proven to be reliable at the lower end of the systolic pressure of less than 40 mmHg which is particularly relevant in patients with severe POAD.

Commonly, toe systolic pressures and TBIs are measured in vascular or research laboratories by trained technicians using nonportable PPG equipment [32]. PPG assesses blood flow by emitting an infrared light that is reflected by the red blood cells in superficial vessels and detected by the transducer. The amount of reflected light corresponds to pulsatile changes and tissue blood volume [32]. PPG does not measure absolute blood flow, but it does provide a functional assessment of perfusion status.

The toe systolic pressure can be measured in the clinical setting using a manual or automatic sphygmomanometer. The automatic sphygmomanometer is electronic, easy to operate, and minimises the impact of observer-subject interaction on the measurement of blood pressure in the clinical setting [33]. The role of the observer in recording the systolic pressure is eliminated and replaced with a digital device programmed to take readings at specific intervals. In comparison the manual sphygmomanometer provides absolute measurements and the units do not require re-calibration. This technique offers more control to the clinician when releasing the device; this is particularly useful as the range for the toe systolic pressure is not wide.

As both manual and automatic testing techniques are emerging to be accessible and recommendations of their use are increasing there needs to be studies investigating both the reliability and validity of these measures [34-36]. The aim of this study was to determine both the intra and inter-rater reliability of the measurement of toe systolic pressure and the Toe Brachial Index in patients with diabetes using a manual sphygmomanometer and PPG.

## Methods

### Participants

Institutional ethics approval was granted by the Faculty of Health Science Ethics Committee at La Trobe University (Human Ethics Application Number FHEC09-90) prior to the study and all participants provided written informed consent. A convenience sample of thirty participants with diabetes was recruited from a university podiatry clinic [37]. Most of the patients who attend this clinic do so to have their foot health status screened and to receive basic foot care. Participants were eligible for inclusion if they were 21 years of age and older and available during the planned time for tests. Participants were excluded if they were unable to lie supine for the duration of the tests, presented with wounds or infection around the testing site and individuals who had a vasomotor condition such as Raynaud's disease.

### Raters

Three podiatrists volunteered as raters. Raters A and B had 1 year and 6 months of clinical experience with the measurement, respectively. Raters routinely took toe systolic pressure measurements with an average of 10 measurements per week in their clinical setting. Rater C was a final year undergraduate podiatry student, who had limited clinical experience. Prior to commencing data collection, all raters undertook a sixty minute training session which allowed them to familiarise themselves with the study protocol and standardised measurement technique. The training session occurred one week prior to data collection.

### Procedures

Participants were provided with pre-test guidelines to reduce the impact of external influences on measurements. This included refraining from tobacco smoking and caffeine intake for at least one hour prior to data collection [15,38]. Prior to measurement each participant lay supine with their legs at heart level for twenty minutes. This was to prevent hydrostatic effects on the pressure reading [8,21,38,39]. Room temperature was measured and maintained at a minimum of 20 to 22°C at both sessions to prevent vasoconstriction of digital arteries [15,21,22].

To determine intra-rater reliability, Toe Systolic Pressure and the TBI were repeated by raters across two sessions [39,40]. Measurement sessions occurred one week apart. To determine inter-rater reliability, independent measurements were taken by three raters on the same group of participants. The time period between the raters tests was approximately 5 minutes. The order in which participants were measured was randomised for both sessions using an Excel random order generator [41]. In order to control bias with respect to the inter-rater analysis, raters were blinded to each others results but not their own.

### Measurement technique

#### Toe systolic pressure assessment

**Figure 1:**
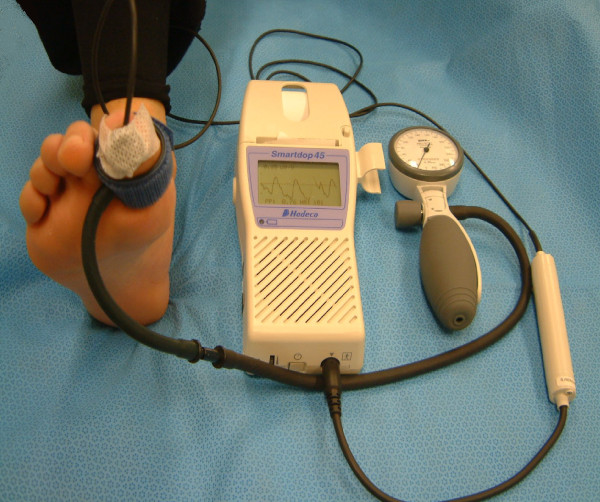
**Measurement of Toe Systolic Pressure using a manual PPG unit (Hadeco Smartdop 45)™**.

Toe systolic pressure measurements were taken with the Hadeco Smartdop 45™ (Figure 1). Initially a 2.5am × 9 cm digital cuff was placed on the proximal aspect of the hallux and the PPG probe was secured onto the pulp of the right hallux with hypoallergenic tape [42]. When a regular waveform was seen on the screen, the sphygmomanometer was pumped up slowly to occlude digital blood flow, to a maximum of 200 mmHg [8]. Upon slow release, the point at which the waveform began to return was regarded as the toe's systolic pressure. Visual and audio representation of the return of the toe systolic pressures were indicated on the PPG unit.

#### Toe Brachial Index (TBI)

The TBI was calculated as the ratio of the toe systolic pressure to the value of the arm brachial systolic pressures as described by Brookes et al [8,21]. Once the value of the brachial systolic pressure and the hallux systolic pressure were obtained, the calculation of the TBI was determined by dividing the toe systolic pressure by the brachial systolic pressure.

### Statistical analysis

Data was analyzed using the Statistical Package Social Science software version 17.0 (SPSS Science, Chicago, Illinois, USA). Data from the right side only of the patient's hallux was collected to satisfy the assumption of independence of data [43]. Data was explored for normal distribution using the Shapiro-Wilks test.

Intra-rater agreements were calculated using the Intra-class Correlation Coefficient (ICC) with model 3, 1. The ICC assesses the strength of linear correlation between two measurements and detects random and systematic error. The 95% Limits of Agreement (LOA) were calculated to assess the level of intra-rater agreement in the related units of measurement [40]. Paired t-test was used to assess for systematic differences in intra-rater data. *P*-values were considered significant at the adjusted alpha of *p *less than 0.01 given there were three comparisons. To establish the average intra-rater ICC across raters A, B and C a form of standardized *z *scores were used. Individual raters' ICC values were transformed to z-scores. The resulting z-scores were averaged, then transformed into *r *values [44].

Inter-rater reliability was evaluated using ICCs (model 2,3) and 95% LOAs [45]. A mean inter-rater 95% LOA was derived from an average of the data, from all raters. A two way repeated measures ANOVA was used to assess for systematic differences between raters. *P*-values less than 0.01 were considered significant given four comparisons. Bland-Altman plots were used to show the differences between two measurements against their mean for the experienced raters (A and B).

## Results

Participant characteristics are reported in Table 2. The majority of participants were older male with duration of diabetes over 10 years.

**Table 2:** Characteristics of study population

Characteristic	Results
Sample size	n = 30
Gender (%)	Male = 57, female = 43
Age (years) ^+^	70.0 ± 8.0
Diabetes type (type 2%)	100
Diabetes duration (years) ^+^	11.5 ± 8.2
Vascular status	Previous history of vascular surgery = 36.7
	Intermittent claudication symptoms = 36.7
	Rest pain symptoms = 3.3
Ankle brachial index^+^	1.1 ± 0.3

Note: Means ± standard deviation^+^

Following the Shapiro-Wilks test, data was explored visually for normality. Data for toe systolic pressures and TBIs appeared to follow a normal distribution.

### Toe Systolic Pressure

Mean and standard deviations for each rater, at session 1 and 2, are shown in Additional file 1. Intra-rater reliability ICCs for Toe Systolic Pressure ranged between 0.83 and 0.89, and the mean 95% LOAs ± 26 mmHg for all three raters (Table 3). For inter-rater reliability, the ICC for session 2 was higher than session 1 at 0.91 and 0.88, respectively (Table 3).

**Table 3:** Intra-class correlation coefficients (ICCs) and the 95% Limits of Agreement (95%LOA) for the intra-rater reliability of the measurement of the Toe Systolic Pressure

	Intra-rater reliability	
	
Rater	**ICC**^**3,1 **^**(95% CI)**	95% LOA (mmHg)
A	0.88 (0.77 to 0.94)	(-23.44 to 26.18)
B	0.83 (0.67 to 0.91)	(-31.56 to 29.36)
C	0.89 (0.79 to 0.95)	(-22.90 to 22.63)
	
Average	0.87 (0.74 to 0.93)	(-25.97 to 26.06)

	Inter-rater reliability	

	ICC^2,3 ^(95% CI)	95% LOA (mmHg)

Session 1	0.88 (0.79 to 0.93)	(-22.91 to 29.17)
Session 2	0.91 (0.85 to 0.95)	(-21.68 to 17.42)

Note: ^+ ^= z-Transformed data; 95%CI = 95% confidence intervals; ICC^3,1 ^= Intra-class coefficient, type3,1; ICC^2,3 ^= Intra-class coefficient, type 2,3.

The paired t-tests for the intra-rater data were not statistically significant at the adjusted alpha level of *p *< 0.01. Similarly, the repeated measures ANOVAs for the inter-rater data were not statistically significant at the adjusted alpha level of *p *< 0.01. Clinically these results suggest error associated with intra and inter-rater data for the toe systolic pressure was random and not a result of systematic differences.

**Figure 2:**
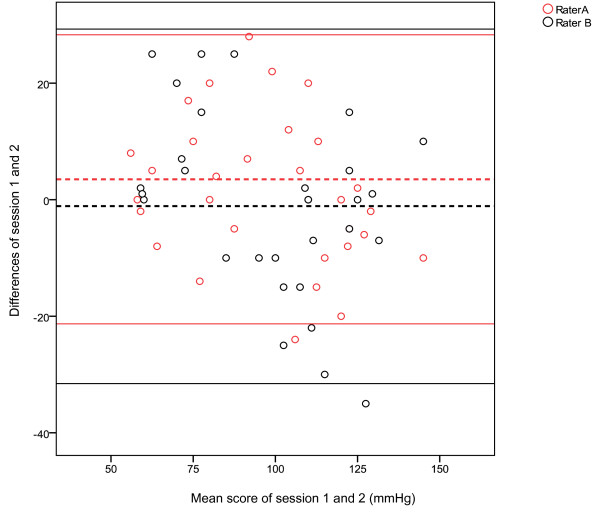
**Bland Altman plots with 95% Limits of Agreement for the measurement of Toe Systolic Pressures for raters A and B**.

Figure 2 illustrates the Bland Altman plots between session 1 and session 2 for the measurement of toe systolic pressures, raters A and B. This figure displays a 95%LOA bias of 3.5 with a SD bias of 12.66 (Lower limit -21.31, Upper limit 28.31) for rater A, which is indicated by a wide LOA. The spread of data for rater B was wider than rater A, with 95%LOA bias of -1.1, with a SD bias of 15.54 (Lower limit -31.56 , Upper limit 29.36).

### Toe Brachial Indices

Mean and standard deviations for each rater, at session 1 and 2, are shown in Additional file 1. Intra-rater ICCs for the TBI ranged between 0.72 and 0.80, however the 95% LOAs ranged between -0.22 to +0.28 and the lower limit of the 95%CI of the ICC was below 0.63 (Table 4). The inter-rater reliability ICC for session 1 and 2 was 0.77 and 0.81, respectively, however again the 95%LOAs were wide relative to the magnitude of the overall measurement (Table 4).

**Table 4:** Intra-class correlation coefficients (ICCs) and the 95% Limits of Agreement (95%LOA) for intra- and inter-rater reliability of the measurement of the Toe Brachial Index

	Intra-rater reliability	
	
Rater	**ICC**^**3,1 **^**(95% CI)**	95% LOA (mmHg)
A	0.72 (0.50 to 0.86)	(-0.24 to 0.32)
B	0.73 (0.52 to 0.86)	(-0.22 to 0.30)
C	0.80 (0.63 to 0.90)	(-0.19 to 0.23)
	
Average	0.75 (0.55 to 0.87)	(-0.22 to 0.28)

	Inter-rater reliability	

	ICC^2,3 ^(95% CI)	95% LOA (mmHg)

Session 1	0.77 (0.62 to 0.87)*	(-0.21 to 0.22)
Session 2	0.81 (0.68 to 0.90)*	(-0.19 to 0.27)

Note: ^+ ^= z-Transformed data; * = statistically significant at *p *< 0.01; 95%CI = 95% confidence intervals; ICC^3,1 ^= Intra-class coefficient, type 3,1; ICC^2,3 ^= Intra-class coefficient, type 2,3.

The paired t-tests for the intra-rater data were not statistically significant at the adjusted alpha level of *p *< 0.01. The inter-rater data ANOVAs showed no statistically significant differences with the adjusted *p *value of <0.01. Two significant differences were found with the TBI data, for sessions 1 and 2, at *p *= 0.02. Post hoc testing using paired t-tests showed that the difference was between rater A and B for both sessions with a mean difference ranging from 0.05 to 0.06. Systematic error to this group of measurements was, if truly present, not clinically significant.

**Figure 3:**
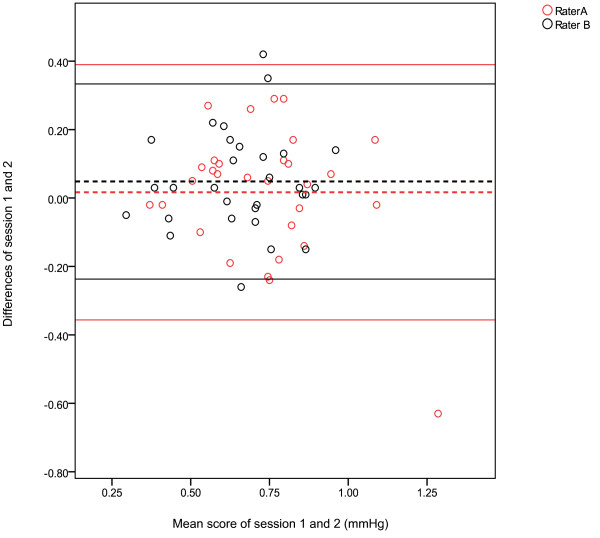
**Bland Altman plots with 95% Limits of Agreement for the measurement of Toe Brachial Indices for raters A and B**.

Figure 3 illustrates the Bland Altman plots between session 1 and session 2 for the measurement of TBIs, raters A and B. This figure displays a 95%LOA, bias of 0.02 with a SD bias of 0.19 (Lower limit -0.36, Upper limit 0.39) for rater A, which is indicated by a wide LOA. The spread of data for rater B was slightly narrower when compared to rater A, this was shown by a 95%LOA bias of 0.05 with a SD bias of 0.15 (Lower limit -0.24, Upper limit 0.33).

## Discussion

The usefulness of a measurement in clinical practice depends to a large degree, on the extent to which clinicians can rely on data as accurate [40]. As the incidence of diabetes escalates so too will the reliance on the use of reliable and valid non-invasive arterial assessment modalities to provide better care to patients. With an increase in interest and the limited usefulness of ABIs in patients with medial calcification, the measurement of the toe systolic pressure and TBI has emerged as a potential useful assessment modality. However, there appears to be few data about the intra and inter-rater reliability of the measurement of the toe systolic pressures and the TBIs using a manual PPG unit (Hadeco Smartdop 45).

Based on ICC values found in this study, the measurement of the toe systolic pressure and TBIs have moderate to good reliability. However, a clinical significant margin of error is evident. This finding has important clinical implications regarding the use of the measurements and interpretation of their output. For toe systolic pressures the 95% LOAs suggest that to attribute a difference in toe systolic pressure to a true change and not measurement error, the observed change must be ±26 mmHg and 30 mmHg when performed by the same rater or different raters, respectively (Table 3 and 4). This is a large range, considering toe pressures are often less than 100 mmHg and in this population may range between 40-90 mmHg. For example, if a toe systolic pressure measurement was found to be 70 mmHg, then the results of this study suggest that we can be 95% confident the true score lies between 40 mmHg and 100 mmHg. In the clinical context this is quite a large error range given that toe systolic pressures are a measurement used for decision making and diagnosis of PAOD.

Similarly, the 95% LOAs suggests that to attribute a difference in TBI to a true change and not measurement error, the observed change must be ±0.28 and 0.22 when performed by the same rater or different raters, respectively. Therefore, these measurements could be inappropriate to use as a screening tool to determine those at risk of developing PAOD as there is a large error range associated with this measurement. This highlights the relevance of further research investigating the conservative nature of the LOAs and whether this statistic is a very conservative judgement of error.

This study has demonstrated that the reliability of these measurements is similar in raters with experience and without experience. The intra-rater ICC values for toe pressures and TBIs ranged from 0.83 to 0.89 and 0.72 to 0.83, respectively. These results are comparable to the findings from the studies by de Graaff et al. [29] and Scanlon et al. However, our study adds to the work of Cloete et al. [30] and deGraaff et al. [28] who only assessed intra-rater reliability.

A further issue to consider when interpreting the study results was the lower limit of the 95% confidence interval of the ICCs. In relation to toe systolic pressures one of the experienced raters (B) showed a lower limit of the confidence interval of the ICC of 0.67 when compared to rater A and C. The lower limit of the confidence interval of the ICC was below 0.70 for both intra and inter-rater reliability of TBIs which could be considered too low to be clinically useful. According to Portney and Watkins 2009 [40], coefficients below 0.75 suggest moderate reliability as a guide. The level of acceptable reliability must be put in context of the patient and pathology under investigation.

Sources of error in reliability studies can be systematic or random. Based on the results from the paired t-tests for the intra-rater data and ANOVAs for the inter-rater data for toe systolic pressures and TBI, the degree of error in the results was mostly random. Random errors occur from unpredictable factors and are harder to correct, as they are unpredictable in direction. Possible sources for error are in relation to the equipment, the rater and the participant. The equipment could have been a possible source of mechanical inaccuracy, placement of the cuff and the PPG probe can affect the measurement if it is not standardised between measurements. Limited experience with the measurement between raters, could have increased the likelihood of simple mistakes such as differences in the control of the release of the manual sphygmomanometer which could have caused inconsistencies in measurements. The physiological status of the blood pressure of the participants may have varied between sessions.

The results of this study need to be interpreted in context of its limitations. A limitation in the measurements proposed in this study is in relation to sample size of both participants and raters. Previous reliability studies have indicated a minimum of thirty participants to be suitable. However, thirty participants and three raters can be considered a small sample size when obtaining adequate power analysis.

The interval between each rater after taking the toe systolic pressure and brachial systolic pressure was short. After measurements were completed, the participant was allowed to rest for 5 minutes in the same position (supine) before the next rater took the measurements. As measurements on each participant within the same session were performed within a short interval this could have caused vasospacity and post occlusion hyperaemia of the digital vessels. The repeated inflation of the digital cuffs could have affected the measurements and contributed to the large range of error.

Experience between raters was minimal ranging from six months to one year. This may be a relative limitation as it is likely to represent the current population of clinicians who utilise these measurements. The use of these measurements is beginning to emerge as part of common practice on patients with diabetes, so it is likely that clinicians would be considered to have minimal experience with the measurements using a PPG unit.

Finally, the results of this study cannot be extrapolated to patients with severe PAOD as the group of participants included in this study did not present with signs and symptoms of severe PAOD. In addition patients were not accurately assessed for the presence and/or severity of PAOD. If any participants had severe peripheral neuropathy or severe PAOD this may have result in irregular patterns of blood flow that could cause differences in measurements [46]. As health practitioners are more likely to assess peripheral blood flow in the presence of ischemia or wound healing, future research needs to be done to investigate the reliability of these measurements in populations with varying clinical presentations such as PAOD and chronic renal insufficiency. As the purpose of this study was to investigate the reliability of this measurement, further research could include a control or comparison group to determine the reliability studies in that group. In addition further development of the vascular assessment technology is warranted.

## Conclusions

This potentially clinically significant margin of error (95% LOA) raises questions about the reliability of using a manual sphygmomanometer and PPG to measure toe systolic pressure and toe brachial index. When assessing patients with PAOD, it is important to consider all other non-invasive vascular assessment options. The context of toe systolic pressures as a non-invasive investigation that may determine intervention as the gold standard could be magnetic resonance imaging (MRI) angiography.

## Abbreviations

ABI: ankle brachial index; ANOVA: analysis of variance; ICC: intra-class correlation coefficient; LOA: limits of agreement; PAOD: peripheral arterial occlusive disease; PPG: photoplethysmography; TBI: toe brachial index.

## Competing interests

The authors declare that they have no competing interests.

## Authors' contributions

MR participated in the design of the study, carried out data collection and statistical analyses. AR conceived the study, participated in the design of the study, reviewed the manuscript and provided academic support throughout the study. BP conceived the study, participated in the design of the study, reviewed the manuscript and provided academic support. All authors read and approved the final manuscript.

## Supplementary Material

Additional file 1 **Mean ± standard deviation (SD) for the measurement of Toe Systolic Pressures and Toe Brachial Indices according to rater and session**. The raw data for the mean ± standard deviation of Toe Systolic Pressures and Toe Brachial Indices according to rater and session. Click here for file
