# Supplementary material for: The reliability of toe systolic pressure and the toe brachial index in patients with diabetes
Source: J Foot Ankle Res. 2010 Dec 22;3:31. doi: 10.1186/1757-1146-3-31 (PMC3020155; doi:10.1186/1757-1146-3-31)
Supplement: Additional file 1 — Mean ± standard deviation (SD) for the measurement of Toe Systolic Pressures and Toe Brachial Indices according to rater and session. The raw data for the mean ± standard deviation of Toe Systolic Pressures and Toe Brachial Indices according to rater and session. [file 1757-1146-3-31-S1.DOC]

# Additional File 1

**Mean ± standard deviation (SD) for the measurement of Toe Systolic Pressures and Toe Brachial Indices according to rater and session**

______________________________________________________________________________________________________________

Rater A Rater B Rater C

______________________________________________________________________________________________________________

*Session 1*

Toe Systolic Pressure (mmHg) 97.87 ± 23.87 98.87 ± 22.90 101.07 ± 24.8

Toe Brachial Indices 0.75 ± 0.19 0.69 ± 0.19 0.71 ± 0.16

*Session 2*

Toe Systolic Pressure (mmHg) 96.50 ± 27.27 99.97 ± 28.73 101.20 ± 25.07

Toe Brachial Indices 0.71 ± 0.20 0.65 ± 0.18 0.69 ± 0.18

______________________________________________________________________________________________________________
